# Rates of Switching Antiretroviral Drugs in a Primary Care Service in South Africa before and after Introduction of Tenofovir

**DOI:** 10.1371/journal.pone.0063596

**Published:** 2013-05-22

**Authors:** Christine Njuguna, Catherine Orrell, Richard Kaplan, Linda-Gail Bekker, Robin Wood, Stephen D. Lawn

**Affiliations:** 1 Desmond Tutu HIV Centre, Institute for Infectious Diseases and Molecular Medicine, Faculty of Health Sciences, University of Cape Town, Cape Town, South Africa; 2 Department of Clinical Research, Faculty of Infectious and Tropical Diseases, London School of Hygiene and Tropical Medicine, London, United Kingdom; University of Pittsburgh, United States of America

## Abstract

**Introduction:**

Antiretroviral changes (single drug substitutions and regimen switches) limit treatment options and introduce challenges such as increased cost, monitoring and adherence difficulties. Patterns of drug substitutions and regimen switches from stavudine (d4T) and zidovudine (AZT) regimens have been well described but data on tenofovir (TDF) are more limited. This study describes the patterns and risk factors for drug changes of these antiretroviral drugs in adults.

**Method:**

This retrospective cohort study included HIV positive, antiretroviral treatment (ART) naïve adults aged ≥18 years who started ART with two nucleoside reverse transcriptase inhibitors (NRTIs) and a non-nucleoside reverse transcriptase inhibitor. Follow-up was censored at first drug change and analysis focused on NRTI changes only.

**Results:**

Between September 2002 and April 2011, 5095 adults initiated ART in Gugulethu. This comprised 948 subjects on TDF, 3438 on d4T and 709 subjects on AZT. Virological suppression rates at 1 year, regimen switching due to virological failure and overall losses to the programme were similar across the three groups. TDF had the lowest incidence rate of drug substitutions (2.6 per 100 P/Ys) compared to 17.9 for d4T and 8.5 per 100 P/Ys for AZT. Adverse drug reactions (ADRs) accounted for the majority of drug substitutions of d4T. Multivariate analysis showed that increasing age, female sex and d4T exposure were associated with increased hazard of drug substitution due to ADRs. Conversely, TDF exposure was associated with a substantially lower risk of substitution (adjusted hazards ratio 0.38; 95% CI 0.20–0.72).

**Conclusion:**

Regimen switches and virological suppression were similar for patients exposed to TDF, d4T and AZT, suggesting all regimens were equally effective. However, TDF was better tolerated with a substantially lower rate of drug substitutions due to ADRs.

## Introduction

Antiretroviral therapy (ART) toxicity leads to drug changes which are especially problematic in resource limited settings where treatment options are limited. Prior to 2009, stavudine (d4T) was widely used as part of first-line ART in South Africa [1]. Despite d4T having good virological efficacy, it has poor tolerability and is frequently associated with peripheral neuropathy and lipodystrophy that may affect up to 21% and 46% of patients respectively [2]. In light of compelling safety data against d4T, the World Health Organization (WHO) in the 2010 ART guidelines recommended replacement of d4T with tenofovir (TDF), a nucleotide reverse transcriptase inhibitor in first-line ART regimens with a better safety profile [3], [4].

In South Africa, use of TDF was phased in around April, 2010 [5]. A disadvantage of TDF, is that it is significantly more expensive than d4T, with an estimated yearly cost for a TDF regimen being $675 compared to $121 for a d4T regimen [6]. However, cost effectiveness analyses using South Africa data suggests that TDF based regimens have almost 12 months more quality adjusted life expectancy compared to d4T regimens [6]. Thus to provide a clear justification for use of this regimen, comparative data are needed on the virological efficacy and rates of toxicity associated with these regimens.

Whereas data on drug substitutions for adverse drug reactions (ADRs) and regimen changes due to treatment failure have been well described for d4T and zidovudine (AZT)[7]–[11], data on TDF are limited, particularly in sub-Saharan Africa where the drug has been in use for a much shorter duration [10], [12]. TDF has been used widely in developed countries since 2002 [13] so most data relating to TDF toxicity are derived from these countries. Drug substitution due to renal impairment ranges from 0% to approximately 2% in studies conducted in developed settings [14]–[16].

In South Africa, TDF is indicated for all new patients starting ART and in those who develop ADRs to d4T or AZT whereas existing patients already receiving d4T or AZT-containing regimens and who have not experienced ADRs are retained on these drugs.

The objectives of this study were to describe incidence rates of drug substitutions and regimen switches due to virological failure among patients receiving regimens containing d4T, AZT or TDF in a large peri-urban area in Gugulethu, Cape Town, South Africa. In addition, to characterize the reasons and risk factors for ADR-related drug substitutions.

## Methods

### Ethics Statement

Data collected for this study received ethical approval from the University of Cape Town research ethics committee, and written informed consent was provided to collect data anonymously for research purposes.

### Setting

The study was carried out at the Hannan Crusaid Treatment Centre (HCTC) in Gugulethu, Cape Town, which has been described in detail previously [17], [18]. This is a dedicated primary health care clinic that has been providing ART to HIV infected adults and children since 2002. The clinic has screened over 8000 HIV positive individuals since inception and by July 2011, 6519 adults had been initiated on ART [19]. The clinic supports a largely low-income community of approximately 420,000 people, with an antenatal HIV-seroprevalence of 24% in 2009 [20], and a TB notification rate of greater than 1500/100, 000 [21].

The Treatment Centre acts as a referral HIV site for surrounding clinics. HIV infected individuals referred to the clinic are predominately ART naïve patients. Prior to 2010, eligibility criteria for ART included adults with a CD4 count of <200 cells/ul or WHO stage 4 disease. From April 2010, the eligibility criteria were revised to include all pregnant women and patients with tuberculosis (TB) patients who had a CD4 count <350cell/ul [5].

Between 2002 and 2008 the first-line ART regimens consisted of either d4T or AZT in combination with lamivudine (3TC) and either efavirenz or nevirapine. From 2010, first line ART at the Hannan Crusaid Clinic in ART naïve adults consisted of TDF, 3TC and either efavirenz or nevirapine. However, patients that had previously started regimens containing AZT or d4T were maintained on these regimens unless toxicity developed.

### Study Population and Design

This was a retrospective analysis involving a review of records of HIV infected adults receiving antiretroviral therapy. The cohort was defined as ART-naïve adults starting first line ART between September 2002 and 30 April 2011. Eligible study participants included HIV positive adults of at least 18 years of age with a minimum of 1 year follow-up by June 2012.

### Data Collection

This study was an analysis of prospectively maintained electronic records from this study cohort. Any missing data was sourced from medical records. The variables collected at baseline included: i) demographic variables including age and sex; ii) clinical and laboratory variables including WHO staging, CD4 counts, viral loads, pregnancy status, creatinine, alanine transaminase (ALT) and haemoglobin; iii) Treatment variables including ART regimen, treatment start and stop date, reasons for treatment change, new ART regimens, outcomes and dates of outcomes.

### Exposure (Determinant) and Outcomes

The primary outcome variable was risk of first drug change. Drug changes were defined as either an individual drug substitution from d4T, AZT and TDF due to ADRs specifically related to that drug or a regimen switch from first-line to second-line treatment due to development of virological failure. All individuals were censored at first drug change and all analyses focused on changes involving d4T, AZT and TDF.

The secondary outcomes were virological suppression and safety of the regimens. Virological suppression was defined as a plasma viral load less than 400 copies/ml after one year of ART. Safety was defined as a binary variable using the following outcomes: death, loss to follow-up, transfer out and alive and in care. Death and loss to follow-up were used as a proxy for poor safety whilst transfer out and alive and on ART were used as a proxy for good safety.

The exposures (determinants) in this study were age at ART initiation, sex, baseline CD4 count, baseline log viral load, baseline haemoglobin, WHO staging, type of ART regimen (d4T, AZT or TDF) and calendar year of treatment start.

### Analysis and Statistics

Data analysis was conducted using STATA version 11 (StataCorp, College Station, Texas). Continuous variables were described using means and standard deviations for normally distributed data and medians and interquartile ranges for variables with a skew distribution. Categorical variables were described using percentages.

Kaplan-Meier analyses were used to measure time to first drug substitutions due to ADRs. A second Kaplan-Meier analysis of time to loss to follow-up/death (programme losses) was computed to assess safety of the regimens. The risk factors associated with drug substitutions due to ADRs were modeled using Cox Proportional Hazards model. The model included: age at ART initiation, sex, baseline CD4 count, baseline log viral load, baseline haemoglobin, WHO staging, type of ART regimen and calendar year of treatment start. A p value <0.05 was used to denote significance.

## Results

A total of 5106 records were identified from ART naïve patients who started ART in the Gugulethu township clinic between September 2002 and 30 April 2011. Eleven records were found to be duplicates and were excluded. The remaining 5095 subjects met the eligibility criteria were and were included in the analysis.

### Demographic and Clinical Characteristics

Overall, the median duration on ART was 2.7 years. However, the median exposure to TDF (1.6 years) was much shorter than for AZT (3.1 years) or d4T (3.4 years) reflecting the later introduction of TDF into the first-line ART regimen **(** **Table 1** **).** The median ages of the patients starting treatment and proportions who were females were similar across the TDF and d4T group. Patients starting AZT tended to be younger and were predominately female (90.1%) **(** **Table 1** **).** Overall, compared to patients starting either AZT or TDF, those starting regimens containing d4T had more advanced disease, as reflected by lower median CD4 counts, higher log viral loads and a greater likelihood of having WHO stage 3 and stage 4 disease.

**Table 1:** Demographic and clinical characteristics of ART naïve adults (n = 5095) started on ART in Hannan Crusaid Treatment Centre.

Patient characteristics	Subjects on zidovudine(n = 709) (%)	Subjects on stavudine(n = 3438) (%)	Subjects on tenofovir(n = 948) (%)
Age (years)- median [IQR]	30.83 [26.80–36.23]	34.44 [29.62–40.94 ]	34.41 [29.35–41.04 ]
**Sex**			
Female	639 (90.13%)	2110 (61.37%)	612 (64.56%)
**WHO Stage**			
Asymptomatic (stage 1&2)	410 (57.83%)	727 (21.15%)	418 (44.09%)
Symptomatic (stage 3&4)	292 (41.18%)	2,700 (78.53%)	492 (51.90%)
No information	7 (0.99%)	11 (0.32%)	38 (4.01%)
**Pregnant**			
Pre-ART	340 (47.95%)	57 (1.66%)	68 (7.17%)
On ART	1 (0.14%)	19 (0.55%)	1 (0.11%)
No information	368 (51.90%)	3,362 (97.79%)	879 (92.72%)
**NNRTI/PI based**			
Efavirenz	183 (25.81%)	2,360 (68.64%)	838 (88.40%)
Nevirapine	523 (73.77%)	1,075 (31.27%)	110 (11.60%)
Liponavir/ritonavir	3 (0.42%)	3 (0.09%)	0 (0%)
Median ART duration (years)- median [IQR]	3.10 [1.40–4.70]	3.40 [1.50–5.70]	1.60 [1.20–1.90]
Baseline log viral load (copies/ml)- median [IQR]	4.60 [4.08–5.02]	4.94 [4.51–5.35]	4.65 [4.16–5.15]
Baseline CD4 count (µmol/l)- median [IQR]	139 [89–189]	103 [49–164]	153 [81–215]
Baseline haemoglobin (g/dl)- median [IQR]	10.7 [9.80–11.80]	11.1 [9.70–12.40]	11.64 [10.2–12.90]
Baseline ALT- median [IQR]	19 [12–29]	25 [17–37]	19 [14–30]
Baseline creatinine (µmol/l)- median [IQR]	72 [57–88]	75 [62–91]	64 [54–74]

IQR- Interquartile range; ART- antiretroviral therapy; NNRTI- Non-nucleoside reverse transcriptase inhibitors; PI-Protease inhibitor; ALT- alanine transaminase.

### Drug Changes

The overall total person time of follow-up was 10,875.9 person years and the cumulative person-years of exposure to each drug was shortest for TDF (1330.9 person-years) and longest for d4T (7875.4 person years) **(** **Table 2** **).** Similarly, the median time to first drug change was significantly shorter for those in the TDF group (1.5 years) compared to those in the AZT (2.0 years) or d4T (1.8 years) groups (p<0.001).

**Table 2:** Numbers and rates of d4T, AZT and TDF first drug changes during follow-up.

Type of drug change	Subjects on zidovudine (n = 709)	Subjects on stavudine (n = 3438)	Subjects on tenofovir (n = 948)
Median time (years) to first drug change [IQR]	2.0 [0.6–3.8]	1.8 [0.8–3.3]	1.5 [1.0–1.9]
Total time exposed (person years)	1669.6	7875.4	1330.9
Total drug changes	189 (26.7%)	1597 (46.5%)	73 (7.7%)
Rate of total drug changes (/100PY)	11.3 (95% CI: 9.8–12.9)	20.3 (95% CI: 19.4–21.2)	5.5 (95% CI: 4.3–6.8)
Drug substitutions	142	1409	35
Rate of drug substitutions (/100PY)	8.5 (95% CI: 7.2–9.9)	17.9 (95% CI: 17.1–18.8)	2.6 (95% CI: 1.8–3.6)
Regimen switch	47	188	38
Rate of regimen switches (/100PY)	2.8 (95% CI: 2.1–3.7)	2.4 (95% CI: 2.1–2.7)	2.9 (95% CI: 2.0–3.9)

IQR- Interquartile range.

Overall, use of TDF was associated with a much lower proportion of patients undergoing drug changes (n = 73/948; 7.7%) compared to those receiving AZT (n = 189/709; 26.7%) or d4T (n = 1597/3438; 46.5%) **(** **Table 2** **).** Compared to those receiving TDF, the rates of drug changes (substitutions and regimen switches combined) were approximately two-fold higher among those receiving AZT and four fold higher among those receiving d4T (5.5/100PYs [95% CI:4.3–6.8] versus 11.3/100PYs [95% CI: 9.8–12.9] versus 20.3/100PYs [95%CI: 19.4–21.2]) respectively.

While rates of regimen switches and drug substitutions (comprising total drug changes) were similar in the TDF group, drug substitutions accounted for a larger majority of drug changes among those receiving d4T or AZT **(** **Table 2** **).**

Viral load suppression rates were similar for the three different drug exposure groups, ranging between 89% and 90% after 1 year of ART (data not shown). Similarly, rates of regimen switch were comparable across all three exposure groups suggesting that similar rates of virological failure were associated with these regimens, ranging between 2.4 and 2.9 switches/100PYs **(** **Table 2** **).** In contrast, rates of individual drug switch differed markedly with d4T having higher rates of drug substitution rates (17.9/100PY; 95% CI: 17.1–18.8) compared to TDF (2.6/100PY; 95% CI: 1.8–3.6) and AZT (8.5/100PY; 95% CI: 7.2–9.9) **(** **Table 2** **).**

### Reasons for Drug Changes

Overall, a majority of individuals had a single reason for drug changes, with a small proportion of subjects having more than one reason recorded. Seventy-three subjects on TDF experienced a drug change, with a total of 77 recorded causes; 189 subjects on AZT experienced a first drug change, with a total of 192 recorded causes; 1597 subjects on d4T had a drug change with a total of 1623 recorded causes **(** **Table 3** **)**.

**Table 3:** Reasons for drug changes due to d4T, AZT and TDF at any time during ART (1 change may occur due to more than one reason).

Reason for drug change	Zidovudine (n = 192)	Stavudine (n = 1623)	Tenofovir (n = 77)
Side effect	58 (30.2%)	1202 (74.1%)	16 (20.8%)
Regimen switch due to virological failure	47 (24.5%)	188 (11.6%)	38 (49.4%)
Pregnancy related	62 (32.3%)	145 (8.9%)	5 (6.5%)
Tuberculosis	4 (2.1%)	8 (0.5%)	16 (20.8%)
Defaulted treatment	12 (6.3%)	39 (2.4%)	1 (1.3%)
Hepatitis B	2 (1.0%)	6 (0.4%)	0 (0%)
Other	5 (2.6%)	21 (1.3%)	1 (1.3%)
Reason not stated	2 (1.0%)	14 (0.9%)	0 (0%)

ADRs accounted for a much smaller proportion of TDF changes (20.8%) compared to AZT (30.2%) or d4T (74.1%). Pregnancy (primarily women in the post-natal period) was associated with numerous AZT substitutions, (32.3%) but smaller proportions of d4T substitutions (8.9%) or TDF substitutions (6.5%). Drug substitution in relation to tuberculosis was uncommon in d4T and AZT group but occurred more frequently in TDF group **(** **Table 3** **)**. This could be due to the practice of not prescribing TDF together with aminoglycosides in retreatment or drug resistant tuberculosis, as both drugs are potentially nephrotoxic.

### Types of Adverse Drug Reactions

Renal impairment, lipodystrophy and anaemia were the most frequently occurring ADRs. The ADRs most commonly resulting in drug substitution were anaemia in those receiving AZT, lipodystrophy in those receiving d4T and renal impairment in those receiving TDF **(** **Table 4** **)**. Cumulatively, 18.1% (623/3438) required drug substitutions due to d4T-related lipodystrophy compared to 4.2% (30/709) from AZT associated anaemia and 1.2% (11/948) from TDF associated renal impairment. Within the sub group of individuals requiring drug substitution due to ADRs only, just over half of subjects 623 (51.8%) switching d4T did so due to lipodystrophy, 30 (51.7%) patients substituting AZT did so due to anaemia and 11 (68.8%) of patients substituting TDF did so due to renal impairment **(** **Table 4** **)**.

**Table 4:** Types of adverse drug reactions resulting in drug substitution in d4T, AZT and TDF (denominator = number of patients with drug substitution due to side effects from table 3).

Adverse drug reaction	Zidovudine (n = 58)	Stavudine (n = 1202)	Tenofovir (n = 16)
Lipodystrophy	12 (20.7%)	623 (51.8%)	0 (0%)
Peripheral neuropathy	7 (12.1%)	454 (37.8%)	0 (0%)
Renal impairment	0 (0%)	0 (0%)	11 (68.8%)
Anaemia	30 (51.7%)	6 (0.5%)	5 (31.3%)
Hyperlactataemia	0 (0%)	67 (5.6%)	0 (0%)
Lactic acidosis	0 (0%)	13 (1.1%)	0 (0%)
Other	9 (15.5%)	39 (3.2%)	0 (0%)

**Figure 1:**
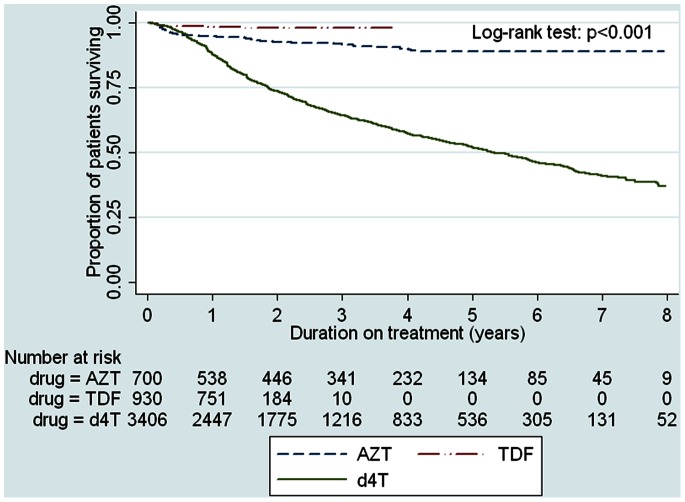
Kaplan-Meier estimates of time to first drug substitution due to ADRs in d4T, AZT and TDF.

**Figure 2:**
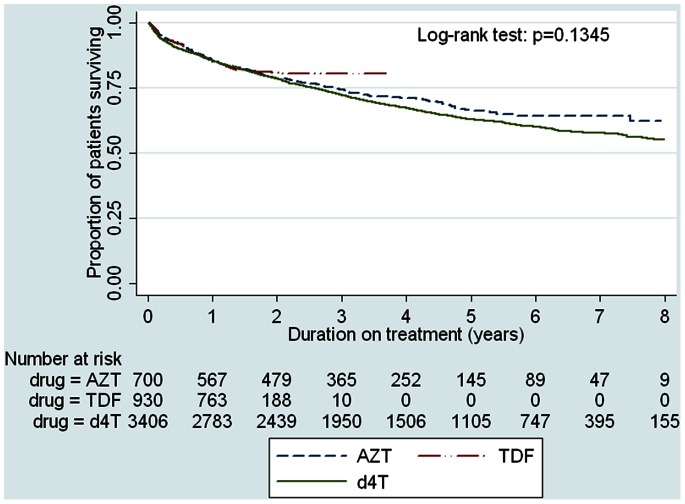
Kaplan-Meier estimates of time to programme losses (loss to follow-up/death) in adults initiated on ART.

A Kaplan-Meier plot, **(** **Figure 1** **),** shows that d4T was associated with the highest risk of drug substitutions due to ADRs over time, whereas TDF was associated with the lowest risk (p<0.001). After 1 year on ART, the proportions of patients who had substituted TDF, AZT or d4T were 1.6%, 5% and 10.5% respectively. By 8 years on ART, 35% (1202/3438) of d4T subjects had a drug substitution due to side effects compared to 8.2% (58/709) subjects on AZT. In contrast, a Kaplan-Meier plot of time to loss to follow-up or death showed no significant difference between the three groups suggesting that losses to the programme were similar across groups **(** **Figure 2** **).**

### Univariate and Multivariate Data Analysis of First Drug Substitution Due to Adverse Drug Reactions

Univariate and multivariate data analyses of time to first drug substitution due to ADRs were done. In univariate analysis, age>40 years, advanced WHO stage disease, lower CD4 count, higher viral load and d4T use were all associated with an increased hazard of drug substitution due to ADRs. Conversely, use of TDF was associated with a lower risk of switching **(** **Table 5** **)**.

**Table 5:** Cox Proportional Hazard model of risk factors for time to first drug substitution due to adverse drug reactions.

	Univariate Analysis		Multivariate analysis	
Variable	Hazard ratio	95% CI	Hazard ratio	95% CI
**Age (years)**				
Age ≤40	**Reference**			
Age >40	1.49	1.32–1.68	1.48	1.30–1.68
**Sex**				
Female	**Reference**			
Male	0.91	0.80–1.02	0.72	0.63–0.82
**Baseline CD4 count**				
CD4<200	**Reference**			
CD4≥200	0.77	0.65–0.91	0.97	0.81–.1.16
**WHO staging**				
Asymptomatic (stage 1&2)	**Reference**			
Symptomatic (stage 3 &4)	1.47	1.29–1.68	0.97	0.84–1.11
Baseline log viral load	1.26	1.15–1.37	1.10	1.00–1.21
Baseline haemoglobin (g/dl)	0.96	0.93–0.99	0.98	0.95–1.0
**ART regimen**				
Zidovudine	**Reference**			
Stavudine	4.99	3.84–6.50	4.89	3.72–6.44
Tenofovir	0.38	0.22–0.67	0.38	0.20–0.72
**Year of ART initiation**				
2002–2005	**Reference**			
2006–2007	0.76	0.67–0.87	0.80	0.70–0.93
2008–2009	0.83	0.72–0.96	0.98	0.84–1.14
2010–2011	0.19	0.14–0.26	1.06	0.72–1.57

In the multivariate data analysis, using AZT as a comparator, d4T regimens were independently associated with substantially increased risk (adjusted hazard ratio 4.89; 95% CI: 3.72–6.44) of substitution whereas the risk among those receiving TDF was substantially lower (adjusted hazard ratio, 0.38; 95% CI: 0.20–0.72) **(** **Table 5** **).**

## Discussion

We describe rates of first drug substitutions due to ADRs and regimen switches due to virological failure in a large cohort of ART naïve adults with 10, 875.9 person years of follow-up. This study reports on the rates of drug changes over a ten year period (2002–2012) that spanned the introduction of TDF based ART in South Africa. We observed that TDF was the best tolerated drug with the fewest drug substitutions due to ADRs. All three regimens appear equally effective with similar rates of virological suppression at 1 year on ART and similar regimen switch rates due to virological failure.

Overall, TDF based regimens had a lower drug substitution rate compared to d4T and AZT. Our results are similar to data from a Zambian cohort as well as a cohort of treatment naïve adults followed up over 7 years in Johannesburg, South Africa [10], [22] Toxicity was a common reason for drug substitution across the three patient groups. We observed that TDF was the best tolerated drug with fewer drug substitutions attributed to toxicity, whilst d4T based regimens were the least tolerated. This trend has also been reported in other resource limited settings as well as in developed settings [10], [15], [22], [23] although our study reported higher proportions of drug substitutions due to ADRs, possibly due to the longer follow up period in our study compared to previous studies or differences in clinical practice.

Lipodystrophy and peripheral neuropathy were the two major toxicities resulting in d4T drug substitutions, whilst anaemia was the most common ADR resulting in drug substitution in AZT as reported by other studies [8], [9], [11]. However, we found a substantially higher proportion of patients required d4T substitution due to ADRs compared to previous studies. This may be a function of longer follow-up and/or tendency to conserve first line regimens in other settings. Compared to d4T and AZT toxicities, TDF was more commonly associated with renal impairment although only 11 (1.2%) patients substituted TDF due to this ADR. Other observational and experimental studies have reported similar low rates of TDF substitution due to renal impairment [12], [14].

Virological suppression rates at one year were similar for all three regimens, suggesting similar short term virological efficacy. By 9 years on ART, few patients from the cohort (5.4%) had a regimen switch due to virological failure, and rates of regimen switching for virological failure were similar for all three regimens. The overall low rates of regimen switching may reflect reluctance of clinicians to switch patients to second-line regimens due to limited treatment options. Alternatively, the regimen switch rates reported in this study were an underestimation of the true virological failure rates as they excluded patients who failed to switch to second-line therapy due to programme losses. Nevertheless, our findings are similar to other studies from resource limited settings [11], [24], [25]. Kaplan-Meier plots showed that losses to the programme (death/loss to follow-up) were comparable across groups, a finding that has been reported in other cohorts [10], [26]. This further underscores the benefit of TDF as a first line agent as it has a better safety profile and equal effectiveness to d4T and AZT.

Multivariate analysis showed that d4T and AZT regimens were associated with an increased hazard of drug substitution due to ADRs compared to TDF based regimens. This further highlights the better safety profile associated with TDF compared to other NRTIs (d4T and AZT). These findings have been reported in both developing settings and first world settings [10], [16], [22]. Increasing age is a known risk factor for ADRs such as lipodystrophy, peripheral neuropathy and renal impairment [3]. In adjusted analysis, female sex and age>40 years were also associated with an increased hazard of drug substitution. Female sex has been identified as a strong predictor of drug substitution in multiple studies [9], [10], [22]. In contrast, data on the association between increasing age and drug substitution are less consistent, with some studies reporting no association whilst others reporting a decreased risk [10], [22]. A large number of individuals required drug substitution due to d4T-induced lipodystrophy and peripheral neuropathy which may explain the association between increasing age and drug substitutions.

Study limitations include the fact that the analysis was restricted to time to first drug change only, thereby underestimating the true number of overall drug changes. Second, data was only available for approximately two years for TDF regimens compared to nine years for AZT and d4T regimens, so possibly underestimating TDF drug changes in the long term. Third, TDF use was preceded by a change in ART guidelines; hence some of the differences observed may be confounded by changes in ART policy and clinical practices. Nonetheless, this study is based on a robust dataset, with a large sample size, long follow-up period and minimal missing data in the described variables.

In conclusion, rates of virological suppression and regimen switching due to virological failures were similar for patients exposed to TDF, d4T and AZT, suggesting these regimens were equally effective. However, TDF was better tolerated with a substantially lower rate of drug substitutions due to adverse drug reactions. These findings support the national policy to include TDF in first-line ART regimens. Further studies with longer durations of follow-up will be required to determine the long term safety and effectiveness of TDF.
